# Fabrication of Bipolar Plates from Thermoplastic Elastomer Composites for Vanadium Redox Flow Battery

**DOI:** 10.3390/polym14112143

**Published:** 2022-05-25

**Authors:** Kannika Onyu, Rungsima Yeetsorn, Jeff Gostick

**Affiliations:** 1 Department of Industrial Chemistry, Faculty of Applied Science, King Mongkut’s University of Technology North Bangkok, Bangkok 10800, Thailand; kannika_kmutnb@hotmail.com; 2 Materials and Production Engineering, The Sirindhorn International Thai-German Graduate School of Engineering, King Mongkut’s University of Technology North Bangkok, Bangkok 10800, Thailand; 3 Department of Chemical Engineering, University of Waterloo, Waterloo, ON N2L 3G1, Canada; jgostick@uwaterloo.ca

**Keywords:** bipolar plate, thermoplastic vulcanizate composite, pyrolytic graphite sheet, woven-carbon-fiber fabric, vanadium redox flow battery

## Abstract

A vanadium redox flow battery (VRFB) is a promising large-scale energy storage device, due to its safety, durability, and scalability. The utilization of bipolar plates (BPs), made of thermoplastic vulcanizates (TPVs), synthetic graphite, woven-carbon-fiber fabric (WCFF), and a very thin pyrolytic graphite sheet (GS), is investigated in this study. To boost volumetric electrical conductivity, WCFF was introduced into the TPV composite, and the plate was covered with GS to increase surface electrical conductivity. Created composite BPs acquire the desired electrical conductivity, mechanical strength, and deformation characteristics. Those properties were assessed by a series of characterization experiments, and the morphology was examined using an optical microscope, a scanning electron microscope, and atomic force microscopy. Electrochemical testing was used to confirm the possibility of using the suggested BP in a working VRFB. The laminated BP was utilized in a flow cell to electrolytically convert V(IV) to V(V) and V(II), which achieved comparable results to a commercial graphite bipolar plate. Following these experiments, the laminated bipolar plates’ surfaces were examined using X-ray photoelectron spectroscopy, and no evidence of corrosion was found, indicating good durability in the hostile acidic environment.

## 1. Introduction

Bipolar plates are an important part of a vanadium redox flow battery, since they provide numerous purposes, while also adding to the cost. A flow field is, commonly, embossed on bipolar plates, which necessitates sophisticated machining [1,2] and delivers electrolytes to the electrode [3]. The flow field design has a direct impact on cell performance, through electrolyte velocity, pressure drop, and electrolyte penetration [3,4], hence, it is critical. A pressure drop in cells influences the energy efficiency of flow batteries [5,6,7], while electrolyte distribution uniformity affects concentration loss [6,8] and voltage efficiency [5]. In order to provide electron pathways, bipolar plates must, also, have a high electrical conductivity [9]. The ohmic loss and voltage efficiency of flow batteries are affected by surface and volume electrical conductivity [10]. The bipolar plate must, also, act as a gasket, to prevent electrolyte leakage and offer mechanical support to the porous electrode structure. Electrolyte leakage may result from mechanical deformation of the bipolar plate, which is troublesome, for obvious reasons. Any attempt to replace the bipolar plate must, therefore, provide the ease of machining high-precision flow channels, excellent electrical conductivity, and strong mechanical strength, all at the same time. These characteristics of BPs directly correspond to cell voltage losses and performance. Equation (1) can be used to describe redox flow battery voltage losses [5,11,12].

E_cell_ = E^0^ − V_act_ − V_ohm_ − V_mass_ (1)

where E_cell_ = actual voltage, E^0^ = standard reduction potential, V_act_ = activation loss, V_ohm_ = ohmic loss, and V_mass_ = mass transport or concentration loss.

The bipolar plate has a variety of consequences on flow battery performance. The cost of the bipolar plates must be reduced, to improve the commercial viability of VRFB. Since they can be manufactured cheaply, including the insertion of complicated flow channels during molding, polymer composites are a viable material for producing bipolar plates [13]. Polymer composites, which have all of the requisite qualities to operate as bipolar plates, must be created without sacrificing other polymer properties such as flexibility, ease of manufacturing, and lightness of weight, while having good mechanical capabilities and chemical stability [3,10,14]. The main barrier for polymer composite bipolar plates is obtaining sufficiently high electrical conductivity. Bipolar plates must have high electrical conductivity on both surfaces (100 S/cm) and volume (the area-specific resistance < 30 mΩ·cm^2^), to transfer electrons to the external circuit [2,13,15]. In past work, a single conductive filler was used to increase electrical conductivity, but the filler content used was more than 80 wt.% [13,16,17]. Such a high mass fraction of conductive filler adversely affected mechanical strength, flexibility, the viscosity of polymer melt, and, hence, the processibility [18]. Several approaches have been investigated to reduce conductive filler content, such as: inserting carbon-fiber papers or fabrics into the polymer matrix via the molding method [19], stacking plain-weave-type carbon prepreg to fabricate the carbon–graphite-hybrid-composite bipolar plate [20], solution casting fluoroelastomer/carbon black composite on carbon-fiber fabric [21] and embedding polyethylene powder and a carbon black mixture in carbon fabric [22]. Surface electrical conductivity is a point on which to concentrate because the contact resistance of each component occurs when the bipolar plate and electrode are compacted together. Accordingly, many techniques have been tried, to increase surface electrical conductivity and decrease contact resistance, via preventing or removing the polymer-rich layer, such as surface polishing via mechanical abrasion and plasma treatment, the soft layer method via absorbing excess polymer by a thin porous film, and surface coating with graphite foil [23]. A graphite sheet or graphite foil has also been used to laminate, on the surface via compression, to increase surface electrical conductivity. Two types of graphite sheets can be used: pyrolytic graphite and expanded-flake-type graphite [24]. The pyrolytic type has a highly oriented structure and Van der Waals bonding in pyrolytic graphite that is stronger than the expanded flake type. To increase the flexibility of the produced bipolar plate, a thermoplastic elastomer has been used as a matrix because of its flexibility and better processibility in a twin-screw extruder and compression molding. Moreover, the flexibility of the thermoplastic elastomer allows it to act as a gasket, to prevent electrolyte leakage in the assembled cell. Thermoplastic vulcanizate (TPV), a type of high-performance thermoplastic elastomer, is a particularly promising option for bipolar plates because it has flexibility, processibility, and high corrosion resistance. It is made by a dynamic vulcanization process and consists of a dispersed phase with a high amount of cross-linked rubber (ethylene-propylene-diene rubber: EPDM), and a continuous phase with a low concentration of thermoplastics (polypropylene: PP) [25,26].

The usage of TPV composite as a bipolar plate material is investigated in this study, along with some of the above-mentioned strategies for enhancing surface and volume electrical conductivity. To increase volume electrical conductivity and reinforce the manufactured bipolar plates, woven-carbon-fiber fabric was put between sheets of TPV composite. A pyrolytic graphite sheet was laminated to the surface of the TPV composites, to improve surface electrical conductivity and reduce contact resistance. Compression molding was used to integrate all of the components, to create a conductive network. The thermal properties of the manufactured bipolar plates were evaluated, to determine the degradation temperature for processibility and the coefficient thermal expansion, which affect bipolar plate thickness, when utilized in an operating flow battery system. Additionally, the electrical conductivities and mechanical properties of the prepared plates were measured, to assess their potential performance in an operating cell. The physical properties such as hardness and density were measured, and the morphology of the surfaces was studied by optical microscope and scanning electron microscope. Lastly, chemical and electrochemical corrosion testing was performed, to investigate the compatibility of the produced plates in an operating flow battery environment. The created bipolar plate’s performance was comparable to that of commercial graphite bipolar plates in all aspects studied and represented a viable alternative to existing commercial options.

## 2. Materials and Methods

### 2.1. Material Selection

Thermoplastic vulcanizate (TPV, Santoprene^TM^ 8211-55, ExxonMobil Chemical, Houston, TX, USA) was chosen as the matrix because it has high chemical stability in vanadium and sulfuric acid. However, the TPV matrix without additional fillers is an electrically insulating material, so electrically conductive synthetic graphite powder (4012, Asbury Carbons, Duluth, GA, USA) was embedded in the TPV matrix during composite preparation. The synthetic graphite contains 99% carbon content, and its typical dimension is 44 × 250 µm, with 1.5 m^2^/g of surface area. To provide additional conductivity as well as enhanced mechanical strength, woven-carbon-fiber fabric (WCFF, HT series, TR30S, Pyrofil, Tokyo, Japan) was used to, simultaneously, achieve volume electrical conductivity and high mechanical properties. Lastly, the polymer composite plates were coated with a pyrolytic graphite sheet (EYG-S121810, Panasonic, Tokyo, Japan), to achieve high surface electrical conductivity.

### 2.2. Thermoplastic Vulcanizate Composite and Bipolar Plate Preparation

**Figure 1:**
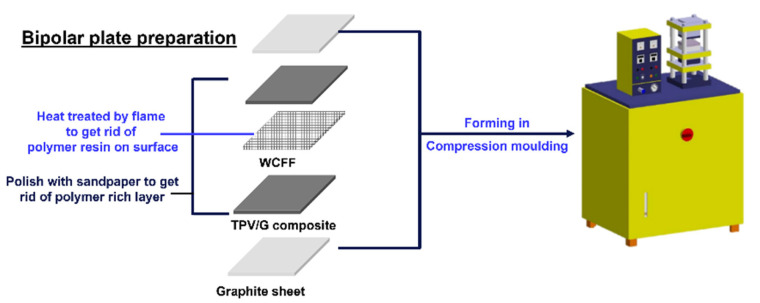
The fabrication process of the prepared bipolar plate.

The TPV composite was prepared by mixing different ratios of TPV and Asbury synthetic graphite. Conventionally, commercial polymer composite bipolar plate uses conductive filler higher than 80 wt.%, to increase electrical conductivity. The goal of this study is to decrease the conductive filler content (<80 wt.%). The TPV/G with different weight ratios (TPV/G: 60/40, 40/60 and 20/80) were dry mixed in a tumbler mixer for 10 min [27]. TPV and G were melt-mixed in a twin-screw extruder (Prism TSE 16TC from Thermo Scientific, Waltham, MA, USA). The temperatures at the feed zone, compression zone, and metering zone were 150 °C, 220 °C, and 240 °C, respectively [28]. The screw speed was 10 rpm. The TPV/G composites were shaped via hydraulic lab presses (CMG 30H-12 ASTM Model B, Carver, Inc., Wabash, IN, USA), at 200 °C and 2000 psi of compressive pressure for 10 min [29,30]. The TPV composite sheet was a polished surface, using sandpaper (grit 600) to remove or to get rid of the polymer-rich layers, which tend to form on TPV-composite surfaces [31]. The fabrication process of the prepared bipolar plate is shown in Figure 1. Two 100 mm × 100 mm slabs of the TPV/G composite were stacked on the top and bottom sides of the heat-treated woven-carbon-fiber fabric. The stacked TPV/G composite and WCFF were sandwiched between pyrolytic graphite sheets on the top and bottom. Finally, the prepared stacks were compressed, as described above.

### 2.3. Characterizations

#### 2.3.1. Thermal Property Testing

Thermogravimetric analysis (TGA) was used, with a heating rate of 10 °C/min under a nitrogen atmosphere (TGA/DSC 3+ from METTLER TOLEDO, Columbus, OH, USA). A temperature range of 30–600 °C was used, to study the composition of TPV and the actual graphite content of TPV composite [32]. The dimensional changes of samples were studied by a thermomechanical analyzer (TMA/SDTA 2+ from METTLER TOLEDO) that tested as a function of temperature in the range of 25–55 °C, allowing for the determination of the coefficient of thermal expansion.

#### 2.3.2. Mechanical Testing

The specimens were tested for compressive strength, according to ASTM D695, in a compression testing machine (QC-506M1, Cometech, Taiwan). The specimen dimension was 12.7 mm × 12.7 mm × 3.0 mm (width × length × thickness). The compressive rate was 1.3 mm/min. The condition for a cyclic compression test force was 0–0.8 MPa and 200 cycles. The density of the samples was measured using a precision balance (PS 1000.R1, RADWAG) at room temperature, following the ASTM D792 standard [33]. The surface hardness of samples was measured with SuperEX durometers (Shore D from e-Asker), following ASTM D2240 [34].

#### 2.3.3. Electrical Conductivity Testing

The surface conductivity of the TPV and laminated bipolar plate was determined by a four-point probe (SP4) and source meter (2400C), according to ISO 3915 [1], to confirm that the graphite sheet outer layer successfully reduced the electrical surface resistance on the surfaces [35]. The area-specific resistance (ASR) of the sample was tested under 0.8 MPa of pressure, by compression. The dimension of the specimens was 100 mm x 100 mm x 1.3 mm. The invented BP specimen was placed between the carbon paper electrode, and they were inserted between the gold-coated copper plate. The current was applied by a DC power supply (PR18-5A, KENWOOD, Tokyo, Japan). The voltage drop of the specimen was measured with a nanovolt meter (2182A, Keithley, Solon, OH, USA).

#### 2.3.4. Morphology Testing

Scanning electron microscopy (SEM, Quanta450, FEI Hillsboro, OR, USA) was used to study the morphology of the samples, to characterize the cross-section of invented BP. The composite specimens were prepared, by freezing with liquid nitrogen to produce a cryodissection. The surface roughness was tested with atomic force microscopy (AFM, Nanowizard3 Ultra, JPK BioAFM, Berlin, Germany). Optical microscopy (HRM-300, HUVITZ, Gyeongju, Korea) was used to study surface roughness, after dipping in vanadium (V) for 7 days at room temperature, for testing acid stability [3].

#### 2.3.5. Corrosion Testing

Chemical corrosion testing was performed, by dipping specimens in vanadium (V) and holding them at room temperature for 7 days. The samples were tested each day for changed mass, altered surface morphology, and changed vanadium concentration in the electrolyte. The vanadium concentration was characterized by a UV-Vis spectrophotometer (SPECORD 210 PLUS, ANALYTIK JENA, Jena, Germany). Samples were tested for electrochemical corrosion, by performing cyclic voltammetry using a potentiostat (VersaSTAT 4, AMETEK, Berwyn, PA, USA). The potential was scanned between 0.0 V to 2.0 V, at a scan rate of 5 mV/s. The system testing consisted of three-electrode cells, with the sample (either commercial graphite BP or laminated BP) used as a working electrode. The diameter of the working electrode was 12 mm. A platinum wire was used as a counter electrode. The reference electrode is Ag/AgCl. The electrolyte solution was 1.6 M VOSO_4_ in 2.5 M H_2_SO_4_ [36]. The functional group on the surface of the sample was characterized by X-ray photoelectron spectroscopy (XPS, AXIS Ultra DLD, Kratos analytical Ltd., Manchester, UK).

#### 2.3.6. Electrolyte Preparation Performance Testing

The laminated BP was used to prepare vanadium electrolyte, in an in-house single cell. A cation exchange membrane (Nafion™ 212, Chemours, Wilmington, NC, USA) was applied for proton transfer and separating the anode and cathode. Carbon paper electrodes (Sigracet SGL 29AA, SGL Carbon, Wiesbaden, Germany) are active sites, where redox reactions occur. The mixed 1.6 M VOSO_4_ (99.9%, Alfa Aesar, Ward Hill, MA, USA) and 2.5 M H_2_SO_4_ (95–97%, Merck, Darmstadt, Germany) were contained in a reactor on the positive and negative sides [37]. The electrolyte was fed into the in-house flow battery cell by a peristaltic pump (BT600-1J/YZ15). The ratio on the positive and negative sides are 50 mL and 25 mL, respectively. The single cell of flow battery was applied voltage at 1.70 V, for preparing V(II) and V(V) by a battery tester (ANQ-T Battery Testing). The reliability of using the invented bipolar plates in a VRFB was investigated by operating the single cell of the VRFB, assembled with the invented composite bipolar plates. The cell operation with commercial graphite bipolar plates (impervious graphite plates (FC-GR347B), Fuel Cell Store) was carried out, in comparison with the performance of the produced bipolar plates. The specification of the VRFB used for these activities is illustrated in Table 1.

## 3. Results and Discussion

### 3.1. Determination of Actual Synthetic Graphite Contents and Thermal Stabilty of the Composites by Thermogravimetric Analysis

In order to define the actual filler content within the final synthetic-graphite-filled TPV, thermogravimetric analysis was performed. Graphite contents were studied in the range of 40–80 wt.%. This graphite concentration range was considered, since 40 wt.% is the percolation threshold [38], and 80 wt.% is the normal graphite content in commercial polymer composite bipolar plate [17]. The percolation threshold is 40 wt.% of graphite that is, initially, connected to graphite particles, to create a conductive network. If the graphite content is less than 40 wt.%, the spacing between graphite particles will grow, causing the electron transfer barrier to shift. The graphite content fraction of 60 wt.% was chosen to be researched, since it is the upper percolation threshold. The electrical conductivity would not be affected, if the graphite content was increased to more than 60 wt.%. The electrical conductivity has altered, slightly, which has an impact on the bipolar plate’s price. During manufacturing of the TPV-based plate, it melted in the twin-screw extruder and was shaped by compression molding. Therefore, the stability of the TPV as a function of graphite content was studied, to be a guideline for determining a window processing temperature. The temperature mixing condition affects the thermal degradation of the TPV/G composite. The TGA result was studied, to find the initial decomposition. The initial decomposition temperature of TPV, TPV/G 40 wt.%, and TPV/G 60 wt.% are 258.86 °C, 260.33 °C, and 272.83 °C, respectively. These results indicate that the processing temperatures stated in Section 2.2. and the VRFB operating temperature reported in Section 2.3.6., will not influence the decomposition of TPV composites. The increasing graphite content affects the initial temperature degradation because graphite provides higher thermal stability than TPV. The different structure of materials affects thermal stability. TPV received the heat. The polymer chain will vibrate and move. Finally, the polymer chain will be degraded. The high fraction of graphite content will increase the initial thermal degradation. Therefore, the operating condition for melt mixing and shaping should be lower than 258.86 °C, to protect against thermal degradation. The TGA results, also, indicated the actual graphite content in the TPV composite: TPV/G 40 wt.% and TPV/G 60 wt.% are 40.05 wt.% and 58.99 wt.%, respectively, as shown in Table 2. The TPV/G 80 wt.% sample could not be processed because the TPV content was insufficient to properly lubricate the graphite particles during extrusion, resulting in an unreasonably high viscosity.

### 3.2. Effect of Structural Composite Design on the Electrical Conductivity of the Bipolar Plates

**Figure 2:**
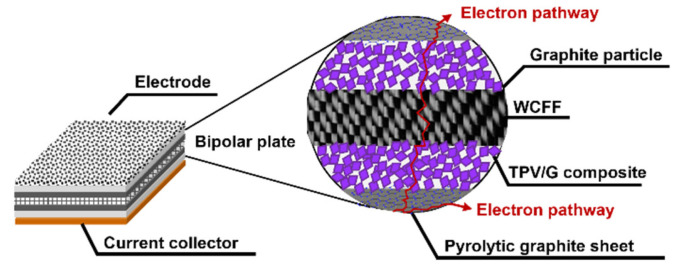
Electrically conductive pathways of the bipolar plates.

Surface and volume electrical conductivity of BPs is necessary for the VRFB performance, since they influence the efficiency of redox reaction occurring during the VRFB operation. The main idea of material selection and design is to create electrically conductive pathways through electrically conductive materials: synthetic graphite and woven-carbon-fiber fabric (WCFF), as shown in Figure 2. Enhancing connections between WCFF and synthetic graphite particles is the strategy to form a conductive network within the polymer matrix, leading to an increased volume of electrical conductivity. The commercial WCFFs were, originally, coated by polymer adhesive, thus, their surfaces have to be burnt to remove the polymer insulator layer that will cause the electrical conductivity increase. The original WCFF had a volume electrical conductivity of 837.20 S/cm, and after flame treatment the carbon fibers were exposed, resulting in increasing the electrical conductivity to 3414.78 S/cm. In terms of the TPV/G composite, once graphite was added to a TPV matrix with 1.13 × 10^−16^ S/cm [29], the electrical conductivity of the mixture was enhanced. The surface and volume electrical conductivities of TPV/G 58.99 wt.% were 6.39 × 10^−6^ S·cm^2^ and 4.04 × 10^−7^ S/cm. Although the electrical conductivity of the TPV/G composite was relatively low, due to a polymer-rich layer coated on BPs’ surfaces generated during a molding process, adding graphite into TPV offered a positive effect on the overall network formation.

The BPs prepared via this compression molding exhibits a smooth surface, since the TPV fills the gap between graphite particles or carbon fibers under the applied compaction pressure. Since most of the conductive fillers get covered under the highly insulating resin, the composite BPs exhibit poor electrical conductivity. The TPV/G composite BPs displayed huge interfacial contact resistance, or area-specific resistance between porous electrodes and BP, causing large overpotential in the VRFBs at high current densities [10,15]. Once graphite particles distribute and disperse in the TPV matrix, there is a high possibility that the graphite particles will attach to the spikes of the WCFF layers. This implies that a continuous path and conductive network emerged. In other words, graphite particles acted as bridges to connect between the pyrolytic graphite sheets and the WCFFs. The electrical current in a flow battery can transport from one graphite layer to the other, through the entire plate. Furthermore, without synthetic graphite particles, the TPV insulator will be an obstacle to electron transfer, since the large insulator layers will be a hindrance to electron hopping. With a gap length of fewer than ten nanometers, electrons can transfer between particles, even if they do not touch [39]. According to this BP design, an alternative method of increasing the surface conductivity was the application of a pyrolytic graphite sheet, with high electrical conductivity around 6675.13 S/cm, to the BP surface. Pyrolytic graphite is, normally, produced from polymer film via thermal heating hydrocarbon, and its graphene sheet is crystallized in a planar direction. Since the pyrolytic graphite sheet was manufactured from a thin polymer film via carbonization and graphitizing, it is thin and has a high surface electrical conductivity. Pyrolytic graphite has the unique features of being thin, lightweight, low density, and flexible. Moreover, the pyrolytic graphite acquires a highly oriented structure, and the Van der Waals bonding in the pyrolytic graphite structure is much stronger than normal graphite [23].

**Figure 3:**
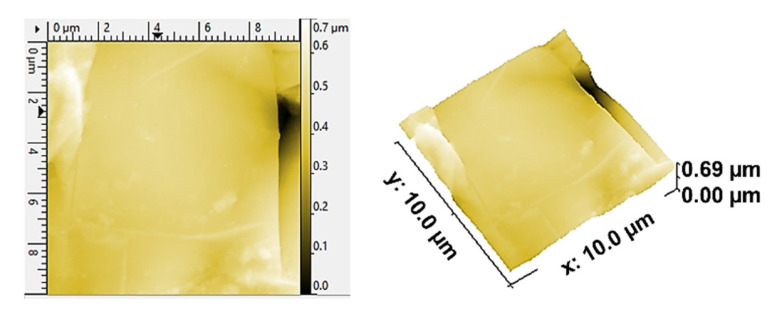
Atomic force microscopy (AFM) image of the laminated BP.

Furthermore, the surface of BP made from TPV/G was polished, to remove the TPV-rich layers for creating contacts between graphite particles dispersing in TPV matrix and the pyrolytic graphite sheet. The surface of the TPV/G 58.99 wt.% was treated with 600-grit sandpaper, and, then, the thickness of TPV/G 58.99 wt.% was reduced from 0.615 mm to 0.590 mm, after treatment. However, the surface and volume electrical conductivities did not change, noticeably, at 6.10 × 10^−6^ S·cm^2^ and 3.64 × 10^−7^ S/cm. The polishing process did affect the smoother surface morphology. The surface morphology is shown in Table 3. The electrical conductivity of the prepared BPs, after laminating with a graphite sheet, was 595.62 S/cm, which is higher than the DOE target (100 S/cm). The area-specific resistance of laminated BP, including embedded WCFF, a laminated graphite sheet, and 58.99 wt.% conductive filler, was 6.46 mΩ·cm^2^. The surface electrical conductivity and area-specific resistance of laminated BP are suitable to use as a bipolar plate in a flow battery. The surface quality of the bipolar plate is an important factor to consider. Surface roughness is classified as the surface quality of a bipolar plate. Surface roughness affects the contact resistance between the bipolar plate and the electrode as well as electrolyte leakage. The optimized surface should be smooth, to prevent electron gaps for electron transfer and protect against the accumulation and penetration of electrolytes on the bipolar plate surface. The surface roughness was studied by AFM. Laminated BP has a mean roughness of 50.70 nm and a root mean square roughness of 75.90 nm (0.0759 µm). The result is shown in Figure 3. The mean roughness of the prepared bipolar plate is lower than the values reported in the literature, where a roughness of 0.46 µm has an area-specific resistance of 24 mΩ·cm^2^ [31]. The prepared bipolar plate, with a roughness of 0.0759 µm, gave a lower area-specific resistance of 6.46 mΩ·cm^2^. Therefore, the low roughness helps to improve contact resistance between the bipolar plate and electrode. The laminated BP has five layers, which consist of two layers of graphite sheet, two layers of TPV/G 58.99 wt.%, and WCFF. The thickness of the graphite sheet is 145.7 µm. The thickness of TPV/G 58.99 wt.% is in the range of 370–430 µm, and the thickness of WCFF is 113.3 µm.

**Figure 4:**
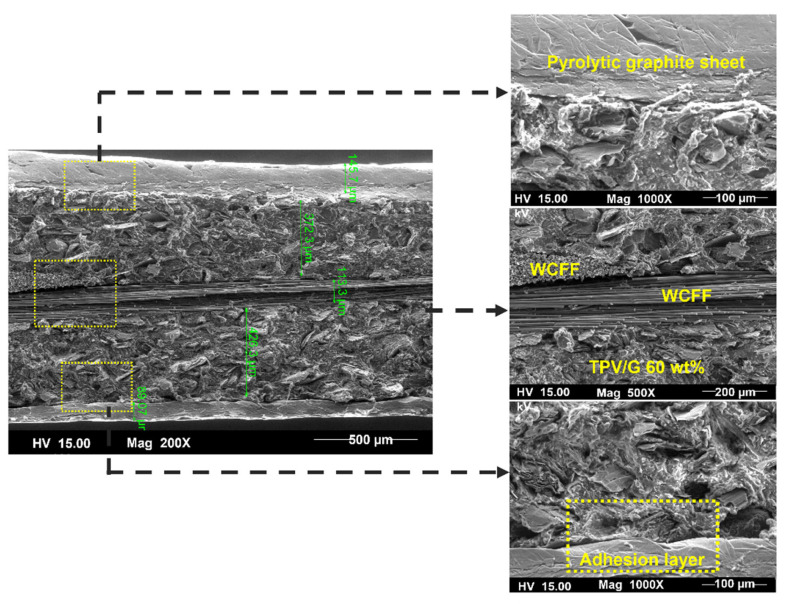
SEM morphology of the laminated BP.

As a result, the interaction between these five layers is crucial. The adherence of each layer was assessed, using SEM images of the sample cross section. Figure 4 shows that there was no visible gap between each layer. In general, contact resistance between material components in the electrochemical devices can be measured, following the resistance test method in fuel cell [40]; nevertheless, the contact resistance between layers inside the main components, as a BP, cannot be directly detected. In this work, the researchers tried to determine the contact resistance of component layers, such as the contact resistance between inserted WCFF sheets using an AFM technique, but the values were not able to indicate the different resistance values. That may be due to the sensitivity of the electrical detector of the AFM. Anyhow, the issue about contacts between each of these five layers are critical, since using electrochemical impedance spectroscopy (EIS) to investigate the interfacial contact resistance in a microstructure of BP will be the future perspective of this research. The impedance data obtained from a simple fit model, related to an equivalent circuit of the VRFB cell, will deliver solution resistance (Rs), charge transfer resistance (Rct), and constant phase element (CPE); these data would be used to investigate the internal contact resistance [41].

For use in a vanadium redox flow battery, the bipolar plate should be lightweight, since many are used in a typical stack. The component weight has an impact on specific energy density (Wh/kg) and specific power density (W/kg). The densities of the prepared TPVs composites were measured as TPV/G 58.99 wt.%, and the laminated bipolar plates are 0.93 g/cm^3^, 1.41 g/cm^3^, and 1.26 g/cm^3^, respectively. To improve mechanical properties, graphite was added to TPV, and graphite has a higher density than TPV. The laminated BP has a density of 1.26 g/cm^3^. Graphite sheet, TPV/G 58.99 wt.%, and woven-carbon-fiber fabric are the three components of the laminated BP. Laminated BP has a lower density than commercial graphite BP, which has a density of 1.99 g/cm^3^. The hardness of laminated BP was 78.5 (shore D), which is lower than the commercial graphite BP (85.0 shore D). The lower hardness will help the flexibility of the surface, to create better seals.

### 3.3. The Relation between VRFB Operating Temperature and the Thermal Expansion of BPs

In this section, a dimension change regarded to the temperature imposed during VRFB operation was observed. A vanadium redox flow battery was technically operated at temperatures ranging from 30 °C to 55 °C, to prevent vanadium precipitation, according to Equations (2) and (3) [42,43,44]. Therefore, the dimension changes of BPs were addressed at a temperature range of 25 °C to 55 °C.

(2VO_2_(H_2_O)_3_)^+^_(aq)_ → VO(OH)_3 (aq)_ + H_3_O^+^_(aq)_ (2)

2VO(OH)_3 (aq)_ → V_2_O_5_·3H_2_O _(s)_ (3)

**Figure 5:**
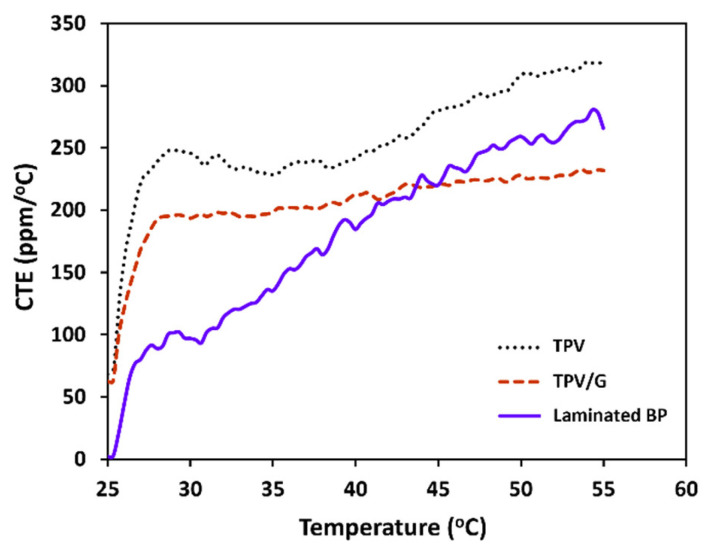
Coefficient of thermal expansion (CTE) of TPV, TPV/G, and laminated BP, as a function of applied temperature.

As a result, thermomechanical analysis (TMA) was used to monitor the changes in the dimension, which occur in materials as the temperature rises [45]. Since cells are operated at elevated temperatures, the TPV, TPV/G composite, and laminated bipolar plate were tested for thermal stability and the coefficient of thermal expansion (CTE). The CTE of TPV composites decreases with increasing graphite content, since graphite acts as a barrier to restrict polymer chain and segment movement. In the laminated BP, woven-carbon-fiber fabric (WCFF) was inserted between TPV composites. WCFF is oriented in an in-plane direction that limits polymer chain movement in laminated BP. Laminated BP has the lowest CTE, when used below 35 °C. The CTE of laminated BP at 25 °C and 35 °C is 2.08 ppm/°C and 140.77 ppm/°C, respectively [46]. Figure 5 shows the outcome: laminated BP is suitable for use in a flow battery.

### 3.4. Effect of Structural Composite Design on Mechanical Properties of the Bipolar Plates

**Figure 6:**
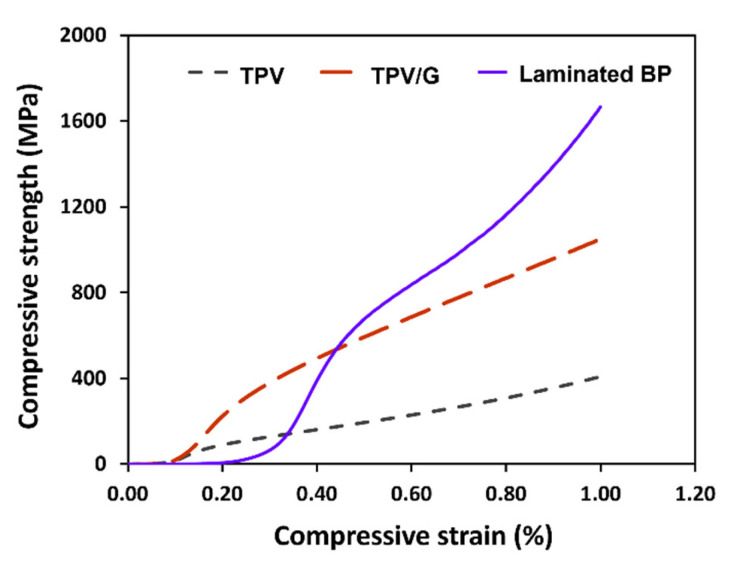
Compressive strength of TPV, TPV/G, and the laminated BP.

There are two approaches to assemble components of VRFB, which are a hydraulic compression machine and assembling with screw bolts and nuts, to protect against electrolyte leakage and decrease the contact resistance of each component. The major force applied to the cell components is a compressive force, thus, the created BPs must endure the compressive force. TPV has viscoelastic properties, which combine with elastic and viscous characters [47] functioning to absorb and distribute the applied stress, while rigid graphite particles and WCFF sheets have a duty to absorb the stress and retard material deformation. At 1% of compressive strength, the laminated composite gave the highest compressive strength (87.42 MPa). As shown in Figure 6, one of the greatest results for compressive strength in commercial polymer composite BPs for flow batteries is 76 MPa. The results show that, when applying force to the combined cell at 0.8 MPa, laminated BP can be employed. DOE technical targets for a composite bipolar plate have a compression strength of 50 MPa [47]. The laminated BP’s compression strength is higher than the DOE target. As a result, the laminated BP’s mechanical properties seem promising for a vanadium redox flow battery.

**Figure 7:**
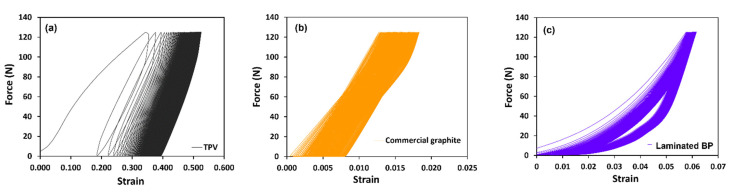
Cyclic force-strain curves for (**a**) TPV, (**b**) commercial graphite BP, and (**c**) laminated BP, at 200 cycles.

The mechanism of component packing, by the compressive force of each position by torque wrench and cell unassembling, was tested using the cyclic loading method. For testing, flow battery components were built and dismantled. When combining cells that hold the compressive force of each bolt position, this part was studied in terms of creep behavior. After using the flow battery, all the components were dismantled. The bipolar plate was studied for relaxation in long-term durability. Due to the general building of plastic strain, cyclic loading can cause fatigue failures [48]. The area of the loading line and unloading line is hysteresis, which corresponds to the absorption of energy, implying an energy loss during the deformation of a viscoelastic material. The graphite BP indicated the lowest strain and lowest area of the hysteresis loop, since the structure of the graphite plate consists of graphite crystalline and is impregnated with phenolic resin. Therefore, graphite acts as a solid elastic, by spring. TPV has viscoelastic behavior that consists of storage modulus and loss modulus. The energy loss is shown in hysteresis, in Figure 7. The strain of laminated BP is in the range of the graphite plate and TPV exhibiting the viscoelastic characteristic. The curve at 200 cycles was smaller than 10 cycles because the polymer chain in prepared BP was arranged to improve stress relaxation.

### 3.5. Chemical Resistance as a Function of Time

**Figure 8:**
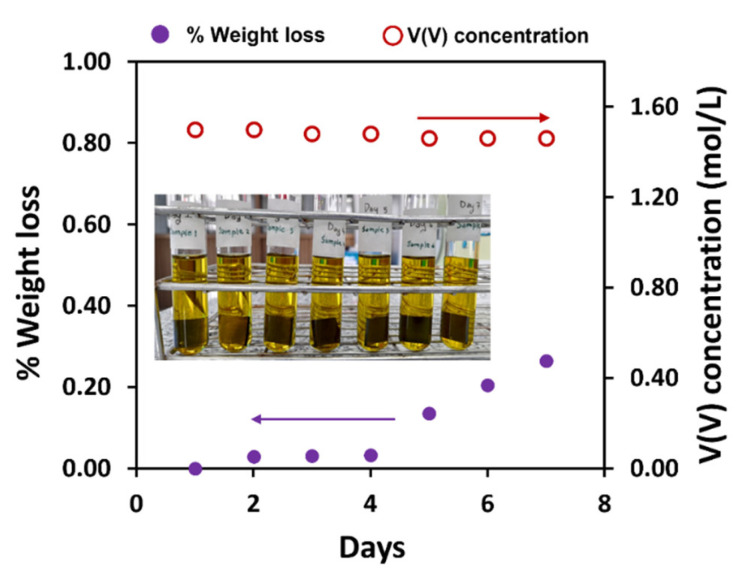
Percentage of weight loss of the laminated BP and V(V) concentration, after testing chemical stability.

**Figure 9:**
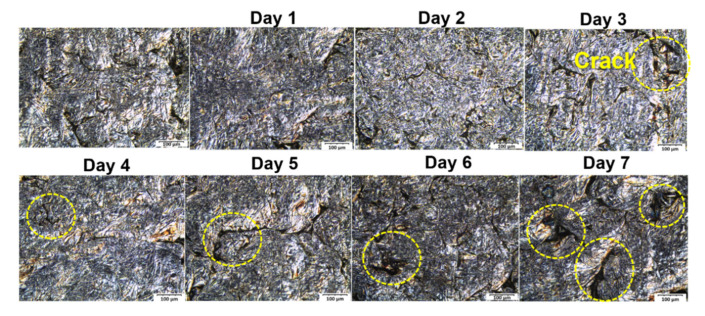
Optical microscopy (OM) images, showing the surface of laminated BP after chemical stability testing.

The chemical stability of a bipolar plate must be very high, given that long lifetimes are desired from these installations. The laminated BPs were tested for chemical stability, by dipping them in vanadium electrolyte, composed of vanadium (V) and sulfuric acid solution, for seven days. The experimental design, for scheduling test duration, was related to the electrolyte preparation. During this step, vanadium electrolytes were maintained for around two days in the VRFB cell, to saturate a membrane and an electrode with electrolyte before testing. This process was manipulated because of improving proton transfer and activating redox reactions on electrode surfaces. The bipolar plates have been contacted electrolytes for two days, thus, researchers intended to test for a longer period of time than the actual time for cell preparation, to study the changed surface morphology and electrolyte. The corrosion issue was studied in a period of electrochemical reaction, occurring during the charging and discharging processes. Vanadium (V) is the most highly oxidizing form of the four vanadium species used in vanadium flow batteries. The weight changes of the laminated BP and vanadium (V) electrolyte concentration were observed every day, for the duration of the test, and are reported in Figure 8. The result showed that weight loss decreases by 0.0008 g or 0.2647%. The concentration of vanadium (V) decreased from 1.50 to 1.46 mol/L, indicating that the vanadium (V) was not actively engaged in an oxidizing reaction. The surface morphology of laminated BP is shown in Figure 9. Some cracks appeared on the laminated BP surface during the aging process. A substantial acceleration in the degradation process was observed after four days of dipping, and this accelerating degradation of the composite material is not a good tendency. The cause of cracking may be owing to the oxidization on the surface of the BP [3]. The oxygen-containing functional groups of the highly conductive graphite sheet may enhance the surface hydrophilicity and facilitate the intercalation of electrolytes into the microcracks and open pores inside the composite BP [49]. This microcracking results in a limited diffusion rate of the vanadium ions in the cracks and pores. Once the cell with cracked BPs is charged, a higher overpotential would be desired. The higher overpotential can convert oxygen-containing functional groups into carbon monoxide or carbon dioxide, and gas accumulation leads to the fracture in BPs. This issue is important for further investigation. The decrease in the electrolyte concentration was, possibly, due to electrolyte diffusion into the surface fracture, and, then, the bulk of the pyrolytic graphite was extracted. The graphite particles may obscure the absorbance of electrolyte solution during the test, via the UV-Vis spectrophotometry technique. That resulted in a decrease in the absorbance and concentration of the electrolyte solution. In general, the plates showed good stability. Exploring the impact of this aging on other properties and over longer periods remains the subject of a future study. Regarding the test, the concentration of VO_2_^+^ was calculated using a calibration curve. The molar absorptivity coefficient was determined, using the absorbance of various VO_2_^+^ concentrations in the range of 0.02–0.10 M, observed at 390 nm of the wavelength, according to Beer’s law dependency.

### 3.6. The Observation of Bipolar Plate Degradation

**Figure 10:**
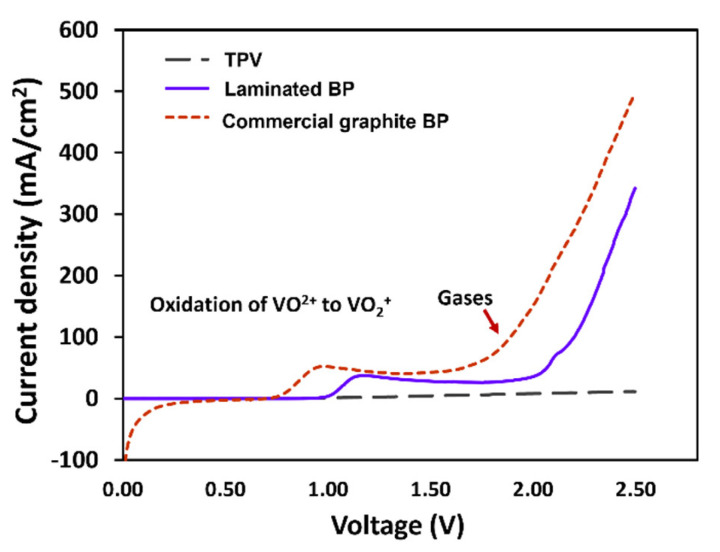
Potentiodynamic polarization curve of laminated BP and commercial graphite BP.

**Figure 11:**
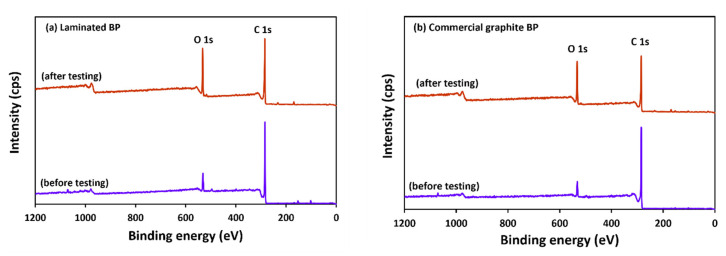
XPS spectra of laminated BP (**a**) and commercial graphite BP (**b**), before and after potentiostatic polarization testing.

The electrochemical stability of BPs was investigated through potentiodynamic polarization curves. The 1.6 M VOSO_4_ and 2.5 M H_2_SO_4_ solutions were used for the test, with the BP specimens as the working electrode, yielding Figure 10. The first anodic peaks of graphite and laminated BP appeared at 0.76 V and 1.00 V, respectively, and this peak indicates that VO_2_^+^ was oxidized to be VO_2_^+^. The apparent anodic peak position confirms the stability properties of BPs, corresponding to the electrochemical corrosion resistance. The composite BP has a higher anodic peak potential than the commercial graphite BP exhibits, while the created BP possessed higher corrosion resistance than that of the commercial graphite BP. Their second anodic peaks occurred at 1.60 and 2.00 V; peaks indicating side reactions on the surface occurred, such as gas evolution, including CO_2_ and O_2_ [50]_._ Since the anodic peak of laminated BP is higher than graphite, it can be concluded that laminated BP has higher electrochemical stability than commercial graphite BP. The wide scan XPS analysis was used to investigate surface change, due to the electrochemical degradation tests. The results are shown in Figure 11a,b. The peaks at ~532 and ~285 eV indicate O1s and C1s, respectively [51]. The O content of graphite, after testing, increased from 6.38% to 20.86%. The O content of laminated BP, after testing, increased from 8.15% to 18.16%. The O/C ratio of graphite and laminated BP, after testing, was 0.27 and 0.23, respectively. Due to lower oxygen content and an O/C ratio derived from a wide scan XPS, the laminated BP has higher electrochemical stability than commercial graphite BP does.

**Figure 12:**
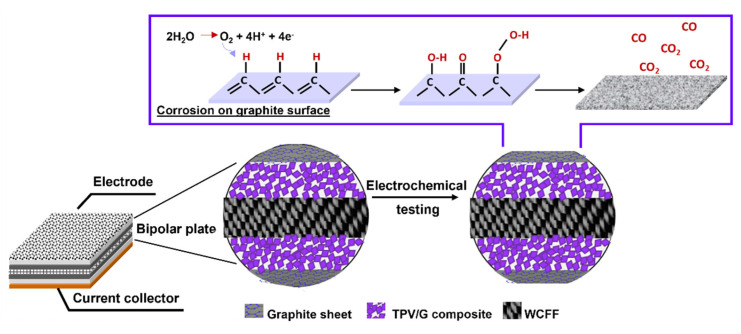
Corrosion mechanism of graphite bipolar plate [36].

**Figure 13:**
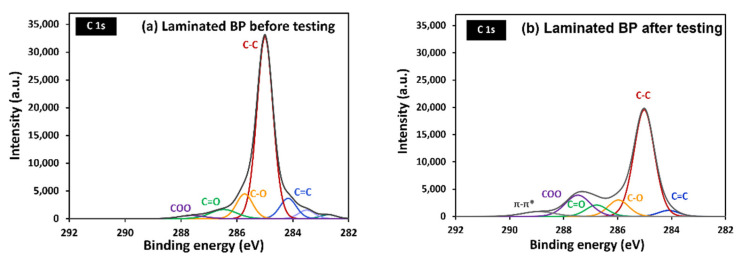
C1s high-resolution XPS of laminated BP, before (**a**) and after (**b**) potentiodynamic polarization testing.

**Figure 14:**
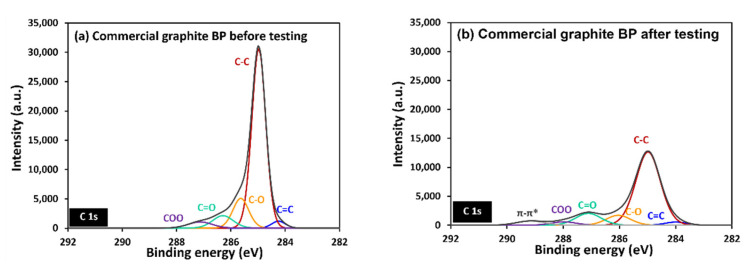
C1s high-resolution XPS of commercial graphite BP, before (**a**) and after (**b**) potentiodynamic polarization testing.

Figure 12 depicts the oxidized carbon atom and the carbon–oxygen functional group on the surface. This indicates that the carbon atom was oxidized on the surface. To identify the function on the sample surface, high-resolution XPS spectroscopy was performed. High-resolution C1s spectra, for laminated BP and graphite BP before and after potentiostatic polarization at 2.5 V, were fitted in Figure 13 and Figure 14, respectively. Six peaks were fitted, including of C-C at 285 eV, indicating the main structure of graphite, C=C at 284 eV, representing unsaturated carbon in graphite structure, and C-O at 286 eV, indicating hydroxyl group. C=O is ascribed a carbonyl group at 287 eV, and a carboxyl group at 288–289 eV [52]. In addition, π-π* transition appears at 289 eV [50]. After performing potentiostatic polarization at 2.5 V, the laminated BP and graphite BP show oxygenated carbon species more dominantly at a higher binding energy tail, as seen in Figure 13b and Figure 14b. This observation supports the corrosion mechanism proposed in Figure 11, that the oxidation of carbon took place on the surface of the laminated BP and commercial graphite BP, during potentiostatic polarization.

### 3.7. The Possibility of Using Laminated for VRFB Application

**Figure 15:**
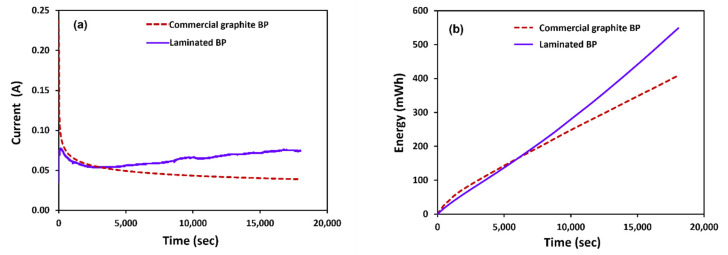
Performance testing of laminated BP, compared with commercial graphite (**a**) current and (**b**) energy, for electrolyte preparation.

Regarding an electrochemical reaction existing during electrolyte preparation, oxidation occurred on the cathode side. The V(IV) was oxidized to V(V) and produced electrons. At the anode side, V(IV) was reduced to V(II). Regarding Figure 15a,b, the flow battery cell consisting of invented bipolar plates offered higher performance, cell current, and generated energy of the charging process (electrolyte preparation) than the cell that used the commercial graphite bipolar plates provided. This result corresponds to the electrical conductivity values of TPV composite bipolar plates (595.62 S/cm) and commercial graphite bipolar plates (8.33 S/cm). The redox reaction generated in both cells at the initial stage was similar in efficiency. After an hour of reaction time, the cell current increased as the reactant conversion increased, resulting in a larger cell resistance due to the higher cell current with constant voltage. The enhanced electrical conductivity of the laminated BPs increased reaction efficiency. The results reveal that laminated BPs outperform commercial graphite BPs, in the preparation of vanadium electrolyte. The average current reflects the oxidation reaction on the electrode. The BP laminates have the highest electrical conductivity because pyrolytic graphite was used to improve surface electrical conductivity. Laminated BPs and commercial graphite BPs had average currents of 64.31 mA and 47.97 mA, respectively. The capacity of laminated BPs is more than that of commercial graphite BPs. Laminated BPs and commercial graphite BPs have capacities of 153.025 mAh and 131.630 mAh, respectively. Laminated BPs and commercial graphite BPs had an energy of 260.157 mWh and 223.781 mWh, respectively. Figure 15 and Table 4 show the results. Although this was not a complete performance validation, it showed that the laminated BPs can be utilized in a vanadium redox flow battery, and the electrical conductivity was adequate. It is worth nothing that the cell current profile, in the initial stage of operating the VRFB assembled with the laminated BPs, was slightly lower than the profile of the cell with the commercial graphite BPs. This stage is, typically, related to the redox kinetics and an activation loss, so further study about the reaction kinetics, via electrochemical techniques such as cyclic voltammetry and EIS, should be carried out. Concerning ohmic loss of the cell polarization, which is the major effect related to material resistance, the cell with laminated BPs showed less ohmic loss because of their high electrical conductivity, which positively impacts the increase in cell current and energy in the second stage. The long durability test can be manipulated to prove this expectation. The issue about a side reaction, corresponded to material degradation, should not be negligible, therefore, it needs to be studied in the future.

In terms of the cost of created BPs, the production cost must be calculated from many parameters such as material cost, labor cost, and utility cost, depending on the types of processing and operating conditions, etc. The cost evaluation will come from a calculation that is based on the current or energy generation, compared to the production cost of the BP. In this section, the price of laminated BP was computed from only the material cost (in lab scale), to be the guideline for enlarging BP production scale. The price of our laminated BP is approximately 26.60 USD/plate (10 cm × 10 cm), while the prices of commercial BPs are 61.00 USD/plate (graphite BP from Fuel cell store, size: 6.3 cm × 6.3 cm) and 42.50 USD/plate (polymer composite BP from MTI Corporation, size: 10 cm × 10 cm).

## 4. Conclusions

Thermoplastic vulcanizate composites were produced, for use as bipolar plates in redox flow batteries, in this work. A plate with adequate volumetric electrical conductivity, low surface resistance, reasonable mechanical strength, high thermal stability, and acceptable chemical durability was created, by combining several concepts from the literature. Compression molding was used, to construct a woven-carbon-fiber fabric in a graphite-filled TPV sheath. To reduce the contact resistance between the bipolar plate surface and the electrode with which it would be in touch in an operational cell, a pyrolytic graphite sheet was laminated to each side of the plate. The electrical conductivity of the layered bipolar plates produced is 595.62 S/cm. The hardness was lower than commercial graphite, allowing the surface to be more flexible and generate better sealing. When utilized to electrolyzed vanadium, the produced bipolar plate exhibited a higher corrosion resistance and performed better than the commercial graphite bipolar plate. Overall, the constructed bipolar plate appears to have a lot of potential for usage in a vanadium redox flow battery.

## Figures and Tables

**Table 1:** The specification of the VRFB single cell.

Specification	Values
Single cell dimension (width × length × height)	126 mm × 126 mm × 67 mm
Active area	30 mm × 30 mm
Temperature range during operation	23–30 °C
Voltage range	1.2 V–1.6 V
Cell current density	40–100 mA/cm^2^
Maximum current	4.9 A
Compression pressure	87 PSI (0.6 MPa), Force is recommended at 116 PSI (0.8 MPa) max.

**Table 2:** Actual filler contents that were determined by thermogravimetric analysis.

Samples	Decomposition Temperatures (°C)			Residual Mass at 600 °C (%)	Actual Graphite Content (%)
	T_i_	T_m_	T_f_		
TPV	258.86	420.00	480.00	9.15	0.00
TPV/G 40 wt.%	260.33	402.50	472.50	45.54	40.05
TPV/G 60 wt.%	272.83	377.50	470.00	62.65	58.99

Note: T_i_ = initial decomposition temperature, T_m_ = middle decomposition temperature, and T_f_ = final decomposition temperature.

**Table 3:** Surface morphology of unpolished surface and polished surface with sandpaper.

Samples	Magnification	
	10×	20×
Unpolished surface with sandpaper	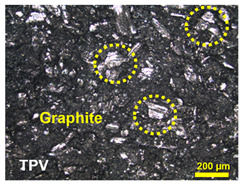	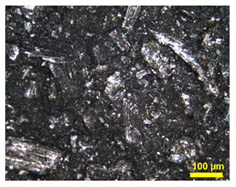
Polished surface, with 600-grit sandpaper	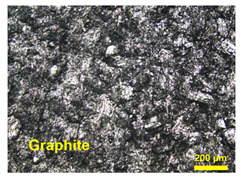	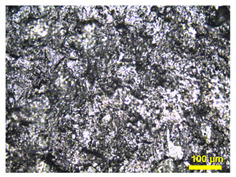

**Table 4:** Performance of BP in vanadium redox flow battery for electrolyte preparation.

Samples	Average	
	Current (mA)	Energy (mWh)
Laminated BP	64.31	260.157
Commercial graphite BP	47.97	223.781

## Data Availability

The data and the code that support the results within this paper and other findings of this study are available from the corresponding author upon reasonable request.
